# On Civilization
*"History of Civilization in England." By Henry Thomas Buckle. Vol. I. London. 1857.


**Published:** 1858-04-01

**Authors:** 


THE JOURNAL

OF

### PSYCHOLOGICAL MEDICINE

AND

MENTAL PATHOLOGY.

APRIL 1, 1858.

### Art. I.—ON CIVILIZATION.*

Whether the human race be one or many as regards its origin, will probably remain for ever a contested point amongst those who depend entirely upon secular history and science for the solution of the question ; and although we hold that the unity of race is the theory in every aspect most accordant with true philosophy, yet it must be confessed that the diversity of condition and development, both physically and intellectually, which is exposed to the investigator at almost every step, would appear almost sufficient to warrant the opinion, that such various habits, manners, customs, and modes of life and thought, could not be the normal characteristics of one species. The Esquimaux, muffled up to the eyes, gorged with many pounds of blubber—the naked inhabitant of Van Diemen's Land, starving on a few oysters or a precarious fish—the New Hollander, rejoicing over his worm which he has just extracted from a piece of rotten wood, or luxuriating over his savoury mess of fern-leaves and pounded ants—the African, broiling his ebony limbs in the burning sun—the Troglodyte, skulking his life away like any other wild beast in his cave ;—these, the extremes of humanity, present almost as strong contrasts amongst themselves as each does to the polished European. This latter, wanting any of the necessaries or luxuries of life, orders it to be "sent in"—the poor Australian "goes out" for it. Let us follow him, fortunate man that he is if he has no friend who has "gone out" before him, in the intention of making his dinner of him. We shall most probably have a weary walk : mile after mile is passed, and no sign of animal life—such, at least, as might be accessible or available for food. His native

* "History of Civilization in England." By Henry Thomas Buckle. Vol. I. London. 1857.

land affords no eatable fruits. He gains the sea-shore, but the sea is now tempestuous, and his expected fish-meal is not to be had; and he probably relieves his gnawing hunger by eating some grubs found in a species of dwarf gum-tree. He may have tracked some bee to its nest, but that is fifty, sixty, or eighty feet up a smooth, branchless tree: hunger is a strong stimulus, and with his rude hatchet he notches the tree with steps, grasping the trunk with his left arm, and so painfully and slowly attains his object. And so his day passes in the search (often unavailing) for food. When night comes, he does not go home, for he has no home to go to; but he puts up a piece of bark or some branches of a tree, to shelter him from the wind; sometimes kindles a fire; and falls asleep, to awake again and resume his weary quest for food. He has no trouble with dress, so that there is nothing to delay his march in search of breakfast. Some day he will be found starved to death on the coast, after an unusually scarce season. He believed in a Koyan and a Potoyan —a good and an evil spirit; but he had but few hopes or fears connected with either.

This is a sad spectacle of degraded humanity, but there are depths still lower, or at least more revolting, than this. Dr. Pickering, speaking of passing through one of the villages in his visit to the Feejee Islands, says—"The male inhabitants were absent at the time, engaged, it was said, in *cooking a man*—a statement which, although it was not doubted, we did not feel particularly desirous of verifying." The same learned writer adds:—"In other parts of the globe, instances of cannibalism have occurred, sometimes from extreme necessity, or as a deed of savage ferocity; and we read of tribes who practise it as a ceremony, religious rite, or even as a *manifestation of affection.** At the Feejee Islands the custom rests on different grounds. It is here interwoven in the elements of society; it forms in no slight degree a pursuit; and it is even regarded in the light of a refinement. Instances are of daily occurrence; and the preparation of human flesh calls into requisition a variety of culinary processes, and is almost a distinct art."

* The Battas of Sumatra are, in comparison with some tribes, a polished people; they have a currency, a regular system of laws and government, and a literature; yet they eat their parents, "and that not so much to gratify their appetite as to perform a pious ceremony. Thus, when a man becomes infirm and weary of the world, he is said to invite his own children to eat him, in a season when salt and limes are cheapest. He then ascends a tree, round which his friends and offspring assemble, and, as they shake the tree, join in a funeral dirge, the import of which is, 'The season is come, the fruit is ripe, and it must descend.' The victim descends, and those who are nearest and dearest to him deprive him of life, and devour his remains in a solemn banquet."—*Moore's Papers on the Indian Archipelago.*

In order fully to appreciate the conditions under which uncivilized humanity is found to exist, we must glance at one or two other tribes, wherein we shall see depicted what some philosophers have considered as "original man." Amongst the "WILD PEOPLE" of Ceram, society can scarcely be said to exist: each family is at perpetual war with all around. They have no houses nor huts, but live and sleep in trees, the branches of which they twine so as to form some defence from their fellows. In the Malay Peninsula there is a forest tribe, called by writers the "ORIGINAL PEOPLE." They live in the deepest recesses of the forest; they never come to the villages, for fear of meeting some one. They neither sow nor plant, but live on fruits and what animals they can take. Their language is understood by no one. They have no king nor chief, but a Puyung, to whom they appeal in disputes. They have no religion whatever, nor any idea of a Supreme Being. When one dies, they bury the head and eat the body.

Dalton thus describes the "WILD PEOPLE OF BORNEO:"—

"Further towards the north are to be found men living absolutely in a state of nature, who neither cultivate the ground nor live in huts; who neither eat rice nor salt; and who do not associate with each other, but rove about the woods like wild beasts. The sexes meet in the jungle, or the man carries away a woman from some campong. When the children are old enough to shift for themselves, they usually separate, neither one afterwards thinking of the other. At night they sleep under some large tree, the branches of which hang low. On these they fasten the children in a kind of swing. Around the tree they make a fire, to keep off the wild beasts and snakes. . . . These poor creatures are looked on and treated by the Dayaks as wild beasts. Hunting parties go out, and amuse themselves by shooting at the children in the trees, the same as monkeys, from which they are not easily distinguished."

The men are all killed, some of the women kept alive, and the children are kept to paddle the canoes, having one foot cut off to prevent escape. It appears that the children cannot be tamed sufficiently to be entrusted with their liberty. "Selgie told me," continues the same narrator, "he never recollected an instance where they did not escape to the jungle the first opportunity, notwithstanding many of them had been kindly treated for years." Such is humanity in one of its aspects.

But it is not alone geographically and ethnically that men widely differ from each other. Marked as are the differences between our populations of Western Europe and the people just described, they are perhaps not more so than are the differences between our present condition and that of our earliest times. It is probable that the early history of some of our most refined European States would, if it could be traced, afford scenes as degraded and revolting as those above noticed. To go no further than our own country: if contemporary annals may be trusted, our condition was not very refined. It seems that our ancestors, or, at all events, the inhabitants of our country about 1900 years ago, were but half-naked savages, painted or stained so as to give them a terrible appearance in war, living chiefly on milk and flesh, scarcely acquainted with agriculture, and their only clothing made of skins. That their manners and customs were of the most barbarous nature, may be inferred from the following passage from "Cæsar's Commentaries:"—"Uxores habent deni duodenique inter se communes, et maximè fratres cum fratribus, et parentes cum liberis. Sed si qui sunt ex his nati, eorum habentur liberi, à quibus primum virgines quæquæ ductæ sunt." The religion of the Island was a superstition of the most frightful character—a Polytheism, each divinity of which was most appropriately propitiated by human sacrifice. In short, we have but to look at the barbarous nations of distant lands in the present times, to find a tolerably accurate rescript of what we were before the Roman invasion.*

This is a consideration full of hope and promise, and one which leads all those who have faith in the high destinies of the human race to inquire earnestly what is the change which has come over our land during these two thousand years? what is the essential difference between us and those who *are* so like what we *were?* and what is lacking to these?

The answer to these questions is ready on every tongue, and is comprised in one word,—CIVILIZATION; and very few, having given that answer, will have any doubts as to the completeness with which the problem has been solved. Yet until the meaning of the word has been defined, and its full comprehensiveness indicated, we shall be very far from having arrived at any practical understanding of the subject.

Before entering upon this investigation, some preliminary inquiry is requisite. We have just seen what man is in his lowest state of development, and the question naturally arises, "Is this state an original one, or is it a fall from some higher condition?" To exhaust the question completely, we should add the third

* There is still a third aspect in which the relations of refined and barbarous life might be viewed—viz., in respect of the fact of the existence, even in the most civilized societies, of large masses of men in an utterly uncivilized condition—degenerate, demoralized beings, of intellects as undeveloped, of morals as low, of passions, vices, and crimes as violent and degrading as any to be found in heathen lands. These constitute the DANGEROUS CLASSES, the rock-ahead of all legislation, the insoluble problem of all society. The subject has been partly discussed in our pages before, and will receive further attention, and, we hope, elucidation, hereafter. We therefore omit all but the most passing consideration of it here.

possibility, as to its being an *advance* from some still lower phase. But inasmuch as some of the races to whom we have alluded seem to differ from the monkeys in little else save in some unintelligible rudiments of articulate speech, and the casual accomplishment of kindling a fire, we may leave this view out of the calculation. The subject, from its nature, admits of no demonstration philosophically; yet the spectacle consequent upon such a hypothesis is too grotesque to be accepted as a probability.

"We must fancy man feeling his way at once to the lowest elements of civilization, and the most elementary conceptions of religion. And as savages make no rapid progress (*some* philosophers say they cannot, and all history shows they do not,) without instruction from *without*, and as by the supposition* primeval man could not have any, it is hard to say how many ages he crawled before he walked, lived on berries and acorns before his first incipient attempts at cookery, yelled his uncouth gibberish before he made (if he could ever make) the refined discovery of an articulate language, and lighted on his first deity in the shape of a bright pebble or an old fish-bone, and was in raptures at the discovery! Or, rather, it is hard to say how the poor wretch ever survived the experiment of any such introduction to the world at all. Some philosophers have defined man as a laughing animal. I am afraid that, on this theory, it was some ages before he found anything to laugh at. It must have been very long before his 'differentia' appeared."

Rejecting, then, the consideration of any possible lower grade of humanity than those described, two theories present themselves, one or other of which must necessarily account for barbarous man. The first is, that he is the unmodified type of man as originally created; "natural progress" not yet having accomplished any part of its mission. The second involves the view that he is fallen from a higher original condition, through the agency of some means internal or external to himself. Even on the supposition of the diversity of man's origin, this must still be an exhaustive division, inasmuch as within historic times nations have progressed from a condition of barbarism like that described, to one of very high civilization; and therefore the developmental condition of these in their earliest times is as much the subject of inquiry, as that of those who still remain in barbarity. Direct evidence of the earliest condition of the human race must, from the nature of the case, either be wanting, or be obtained from a revealed source—that is, from an external revelation. The legends which each nation possesses as to its origin are invariably simply fabulous. Therefore to determine man's

* Viz., that of "*natural progress*," which is one of the dogmas of Mr. Buckle's book. The reason for the quotation, which is from the "Defence of the Eclipse of Faith," p. 57, will be apparent in the course of the essay.

primitive state, we have but two methods—reason and analogy, on the one hand; and a reference to those documents which claim to be historical, contained in the earlier chapters of the book of Genesis, on the other. These, rejected by some, and considered by others to be of limited application, are clearly all the direct evidence accessible to us.

What, then, does analogy teach us? Reasoning backwards from the present condition of all civilized countries, we find at each step a slight, though definite, advance towards the condition of savage life, until we arrive at some such condition as that of our own country two thousand years ago. At such a point, authentic records are in general wanting, and our mode of arriving at a knowledge of the previous state, is by theoretically continuing the retrogression from all the arts of civilized life,—a process which will undoubtedly sooner or later bring us to a perfectly wild state. Were this legitimate, we could have no doubt whatever as to man's primeval condition; but besides appearing almost like an assumption of the very question at issue, we are met by two important considerations which seem to cast some discredit on the results, and therefore upon the method.

The first is, that however far we pursue our researches backwards, so long as authentic history is our guide, we find evidences of civilization somewhere—in some nation or people. This of itself would, of course, prove nothing more than that this nation had worked out its own progress earlier than its neighbours; but the second is of more importance. We may safely assert that the history of every civilization of which the history can be traced, is connected with that of its intimate communion with some other more highly civilized people, either in trade, commerce, or war; and that we have no authentic records of a true civilization originating amongst and being completed by one people, unassisted by any other. If analogy, then, be good for anything in such an investigation as the present, it would lead us to infer that the first forms of civilization were not spontaneously originated amongst a people as barbarous as the "*original people*" of Borneo. We should, then, naturally conclude that somewhere or other a higher type of humanity had always existed; and, as a logical consequence, that the *earliest* type was higher, and that these men, supposed to be "in a state of nature," were really fallen *from* a state of nature through some moral or physical agency, or both combined. Let us hypothetically attach even no more weight to the documents alluded to than as a theory to account for the present condition of the human race; even then we shall find it much more universally applicable than any other yet propounded. Let us suppose that there is one family, consisting of several members, possessing some knowledge of useful arts, skilled to some extent in agriculture, and habituated to a pastoral life. Their descendants multiply, and become too numerous for the land in which they dwell; portions of them migrate in different directions, and become modified as to the constitution of their society in accordance with the climate, soil, and natural products of the place where they finally fix themselves. Some establish themselves upon fertile localities, which repay cultivation, and found permanent colonies where their former civilization will to some extent flourish; some adopt a nomadic pastoral life, in which the arts to which they were formerly addicted will become useless, and the remembrance of them gradually disappear. Others, again, will be thrown where the products of the earth are insufficient for their support, and will become hunters; and, in the constant arduous struggle for existence, will still more rapidly lose sight of the laws of civilized life. Lastly, some will be thrown, not from choice, but by accident, where the energies of the whole man will be required to eke out even the most precarious existence from the scanty supply both of vegetable and animal productions; and it is easy to see how the downward course of these will soon bring them almost to a level with the brutes. Add to these natural causes the influence of the excesses, the vices, the passions, the general evil tendencies inherent to a warped moral nature, and we need be at no loss to understand the present degraded phases which humanity so frequently assumes. Such is the theory which we should adopt to account for the varieties of barbarism and civilization.

Nor is this merely a speculative and unimportant question. If the original condition of man be that of utter barbarism and degradation, then the methods by which his elevation from that condition is to be expected must remain merely empirical; nay, even the very possibility of such elevation must remain in each case a matter of doubt until success be achieved. Not so if it be clearly established that such conditions are only the natural results of the operation of degrading moral and physical agencies upon a subject originally capable of higher things. Then, and only then, can we hope that a correct system of moral and physical hygiene will be efficacious in restoring him to his former condition, and capacitating him for further progress. Then, also, should we see a clear indication of the true method of reorganizing and regenerating the barbarous and demoralized tribes or masses which mingle with and corrupt our most civilized communities, and which laugh our present methods to scorn.

As we have seen, the lowest forms of humanity are those where, for his subsistence, man is dependent upon the scanty produce of the chase, fishing, or the natural uncultivated produce of the earth. The first step in his upward course towards civilization is taken when he takes to a pastoral life. Still, without settled habitation, migrating frequently in search of fresh pastures, he endures much privation, and in his life there are but few elements of progress; yet it is one step in advance. The next is a more important one—that of adopting a fixed home, and agricultural pursuits. Hence arise the elements of a social state; for here must flourish to some degree a practice of the arts; there must be a variety of interests, sometimes clashing; a liability to invasion from the nomadic tribes of the surrounding districts, and, in consequence of this, the necessity will be felt for some sort of social combination, marked by more or less law and order; likewise, the society will inevitably divide itself into the governors and the governed. The transition from this to the feudal system is speedy and natural; thence to absolute monarchy; and after that to the highest state of civilization, that "in which security of person and property is firmly established by a just and complete administration of good laws, where public opinion has the greatest influence, and where more happiness is found in the community."*

Such are the external phenomena or the landmarks of advancing CIVILIZATION; but we are yet as far as ever from having ascertained the essential nature of the change, and the agencies which are in operation in its production. The difficulty of giving a scientific and comprehensive definition of the term is acknowledged to be great; perhaps for this reason the writer of the very learned work placed at the head of this essay avoids the attempt, and contents himself with reasoning upon some understood common acceptation of its meaning. This is to some extent convenient, as it permits the actual fact to be viewed in a vague or arbitrary sense, so as to accommodate it to the theories brought forward to account for it, rather than to mould these strictly in accordance with some accurate formula of the fact.

Let us grant, for convenience, that a strictly accurate scientific definition of Civilization is impracticable, and attempt to analyse the meaning of the term in its general and ordinary acceptation.

The etymology of the word will help us but in a very small degree. It gives us the idea of the perfecting of civil and social life, the life of society, the relations of men to each other. But evidently this does not exhaust the question. We will follow M. Guizot in his analysis of what Civilization *is not*.

* Mackinnon's "Hist. of Civilization," vol. i. p. 22.

1. A people whose external life is happy, with well-administered forms of justice, may fulfil the above conditions; but their intellectual and moral existence is in a state of torpor. This is *not* civilization. "We are not without instances of this state of things. There has been a great number of small aristocratic republics, in which the people have been thus treated like flocks of sheep, well kept and *materially* happy, but without moral and intellectual activity. Is this civilization? Is this a people civilizing itself?"—*Guizot.*

2. The material existence may be lower than in the last case, but still tolerable; the intellectual and moral wants are to some extent supplied; they are *meted out* to each individual—no one is permitted to seek for himself. "Immobility is the characteristic of their moral life." The instances of this condition are found amongst the populations of Asia, the Hindoos especially. *This* is not civilization.

3. "I change" (says M. Guizot) "altogether the nature of the hypothesis: here is a people among whom is a great display of individual liberties, but where disorder and inequalities are excessive; it is the empire of force and of chance; every man, if he is not strong, is oppressed, suffers, and perishes; violence is the predominant feature of the social state." Europe has passed through this condition, but it is not civilization.

4. Liberty and equality do not constitute civilization, for such are the elements of savage life.

From these illustrations of what civilization is *not*, we may arrive at some idea of what it *is*. The essential idea seems to be progress and development, moral and intellectual culture, extending and perfecting the social relations: "on the one hand, an increasing production of strength and happiness to society; on the other, a more equable distribution, amongst individuals, of the strength and happiness produced."

But this is not all that is comprised in this word Civilization. In this view, man is considered but as an element of a social community; but he is something more than that; he has his inner life, his faculties, his sentiments, his ideas; these assert their claim to development; and side by side with the perfecting of society, this, the perfecting of humanity, proceeds to complete our conception of a durable civilization. Which of these two must be accepted in the light of *final cause*—whether man perfects his faculties in order to improve his social relations, or whether the improvement in social relation is but the groundwork for the development of the individual: in short, whether man is made to serve society, or society to serve man? These are questions upon which we must not enter. We will conclude this part of our subject with a noble sentiment uttered by M. Royer Collard :—

"Human societies are born, live, and die on the earth ; it is there their destinies are accomplished. BUT THEY CONTAIN NOT THE WHOLE MAN. After he has engaged himself to society, there remains to him the noblest part of himself, those high faculties by which he elevates himself to God, to a future life, to unknown felicity in an invisible world. We, persons individual and identical, veritable beings endowed with immortality,—we have a different destiny from that of states."

But leaving these introductory questions as to the phenomena and essential nature of Civilization, it is time that we inquire into the agencies internal and external to man, which are engaged in its development. As these agencies are treated of most elaborately in the work, the title of which is prefixed to this essay, we propose to give an abstract of the author's views, with such comments as may seem needful, either for purposes of illustration or refutation.

Mr. Buckle has produced the first volume of a work of most extensive and profound research and learning; his theories, however, are startling and extravagant in no ordinary degree. Of those circumstances and institutions which are commonly supposed to influence mightily for good or for evil the fate of nations, he believes in very few. In man's "natural progress" he believes much—in an overruling Providence, not at all; in the power of a people to work out its own beliefs and civilization, he has much faith; but any assistance from without is "a tampering" with this natural progress. In climate, soil, and aspects of nature, there is great virtue and power as to the production of civilization; but *none whatever* in religion, morals, literature, and government.* These, and other propositions of similar import, will receive ample illustration as we proceed in our analysis.

The first chapter is devoted to an exposition of the probability that history is governed by laws as definite as those of physical science; that the elements are more numerous, and, from our imperfect knowledge of them, the results are less easy to predict, and more complex; that the laws of human action are so definite, that if we were fully acquainted with all the elements of character and motive in individuals or communities, we should be able to predict their history with the same certainty that we can predict eclipses of the sun or moon. The doctrines of chance and necessary connexion, of free-will and predestination, are also discussed, and all set aside; Mr. Buckle, so far as we can gather

* *Vide* p. 232.

from his words, believing in nothing but an enchainment of events. Johnson said to Boswell, "Sir, we *know* our will is free, and there's an end on't." This conclusion is opposed on two considerations—first, that perhaps consciousness does not exist as a separate faculty; second, that even granting it to do so, its evidences are not to be trusted! A strange, unpractical way of discussing an important subject. No one doubts the consciousness of the man who, for an infraction of the laws of his country, is condemned to five years' penal servitude; but those who attempt to convince man of his responsibility, and so induce him to avoid such infractions, are met by an incomprehensible metaphysical subtlety. As a specimen of the conclusions to which these arguments tend, we will give one or two quotations:—

"Rejecting, then, the metaphysical dogma of free-will and the theological dogma of predestined events, we are driven to the conclusion that the actions of men, being determined solely by their antecedents, must have a character of uniformity; that is to say, must, under precisely the same circumstances, always issue in precisely the same results. And as all antecedents are either in the mind or out of it, we clearly see that all the variations in the results—in other words, all the changes of which history is full, all the vicissitudes of the human race, their progress or their decay, their happiness or their misery—must be the fruit of a double action; an action of external phenomena upon the mind, and another action of the mind upon the phenomena."—(p. 18.)

"The offences of men are the result not so much of the vices of the individual offender,* as of the state of society into which that individual is thrown. This is an inference resting on broad and tangible proofs, accessible to all the world; and, as such, cannot be overturned or even impeached by any of those hypotheses with which metaphysicians and theologians have hitherto perplexed the study of past events."—(p. 27.)

Such being Mr. Buckle's view of the nature of human responsibility, we feel curious to know how, in proper course, he will justify the formation of a criminal code. Certainly, rewards of virtue and punishments of vice should equally be dead letters, if such be the sole source of action.

The irrefragable proofs above referred to are those derived from statistical tables. It is shown that about the same number of murders, suicides, and other crimes occur annually; and from this it is most strangely assumed as proved, that these crimes are but "the product of the general condition of society, and that the individual felon only carries into effect what is a necessary

* "The moral actions of men are the product *not of their volition*, but of their antecedents."—(p. 29.)

consequence of preceding circumstances. (!) In a given state of society, a certain number of persons must put an end to their own lives. This is the general law; and the special question as to who shall commit the crime depends of course upon special laws, which, however, in their total action, must obey the large social law to which they are all subordinate. And the power of the larger law is so irresistible, that neither the love of life nor the fear of another world can avail anything towards checking its operation ! !"

Is it indeed so? Is human nature a thing only to be summed up by tens, and hundreds, and thousands, in order to investigate and exhaust its conditions? Mr. Buckle speaks of statistics as "a branch of knowledge which, though still in its infancy, has already thrown more light upon the study of human nature than all the sciences put together." Far be it from us to undervalue so important a science; but that it reveals anything whatever of human nature, we dispute entirely. What can the science of statistics know or tell us of the hopes and fears, the cares and compensations, the joys and sorrows, the aspirations and despair, the yieldings and resistances, the passions, desires, and emotions, the motives and hindrances, which in their aggregate influence human action, and *constitute*, so to speak, human nature? Nothing. But after all these have wrought their effect—when the strife is over and the work is done—statistics can step in, and number the slain and the survivors. As well might we try to arrive at the true theory of a military government by counting the slain in its wars; as well calculate the orbit of a planet by counting the *number*, instead of investigating the *nature*, of its aberrations; as well count the acts of parliament by way of ascertaining the principles of legislation, as attempt, by numbering the residuum of conflicting forces, to define the source and principles of human action. The dial of a chronometer tells nothing of the motor force—the balance, the compensations within; it only indicates the general result; and he who would ascertain the laws of humanity from statistics, might also hope to construct a chronometer from the mere contemplation of the dial-plate.

We have dwelt at some length upon this introductory chapter, because in it seems to be contained the key to the whole of Mr. Buckle's strange philosophy, which, if it be intended to mean anything, reduces man to a mere machine, an integral part of society, having neither individuality, free-will, nor responsibility; a view so utterly subversive of all practical legislation, so repugnant to the common sense of mankind, that we cannot too strongly protest against it.

We pass on to notice the effects of climate, soil, and the aspects of nature, upon civilization—a part of the subject in reference to which we find much valuable matter in the work before us, interspersed still with much that is objectionable.

Mr. Buckle does not speculate upon the original state of man, nor his passage through the earliest stages of progress; he takes him up when he has got a fixed home, and argues that the first subsequent step towards civilization must be the accumulation of wealth in the community, so as to leave an overplus after the wants of the people are supplied. For so long as every man is fully occupied in gaining his bread, there can be no leisure for the cultivation of intellectual pursuits, which are (*auct. loquent.*) the sole agents in civilization. But an accumulation of wealth occurring, "now it is that the existence of an intellectual class first becomes possible, because for the first time there exists a previous accumulation, by means of which men can use what they did not produce, and are thus enabled to devote themselves to subjects for which, at an earlier period, the pressure of their daily wants have left them no time."

The first sources of wealth are climate and soil, or, in general terms, the physical peculiarities of the country; and the amount will be determined (1) by the energy and regularity of the labour, and (2) by the returns made by the soil to such labour. The former will be the result of climate, influencing man's power and disposition to work; the latter is dependent on the soil alone. The *extremes* of climate are unfavourable to the regularity and energy of man's work; thus, in Norway and Sweden, from the severity of the winter and the shortness of the days—in Spain and Portugal, from the heat and dryness of the weather—continued agriculture becomes impossible. "The consequence is, that these four nations, though so different in other respects, are all remarkable for a certain instability and fickleness of character; presenting a striking contrast to the more regular and settled habits which are established in countries whose climate subjects the working classes to fewer interruptions, and imposes on them the necessity of a more constant and unremitting employment."—(p. 40.) Mr. Buckle takes an interesting review of the influence thus exerted upon various nations and districts. Civilization in Asia has always been confined to "that vast tract where a rich and alluvial soil has secured to man that wealth without which no intellectual progress can begin." To the north of this district there is a long line of barren country, inhabited by Mongolian and Tartar hordes, who, so long as they remained in this place, never emerged from an uncivilized condition; yet in their migrations arriving at more fertile soils, founded great monarchies in China, India, and Persia, with a civilization equal to that of any ancient people. The Arabs, also, in their own country, were always a rude, uncultivated people; but, conquering Persia in the seventh century, Spain in the eighth, and the Punjaub in the ninth, they founded mighty empires, "built cities, endowed schools, collected libraries; and the traces of their power are still to be seen at Cordova, at Bagdad, and at Delhi." The only civilized part of Africa—*i.e.*, Egypt—is indebted likewise to its soil for its power of elevating itself. Thus, in Asia and Africa, the condition of civilization was a fertile soil. In Europe, however, the case is different: here the climate has more influence than the soil as affecting the energy of man. And hence the primary difference in character between the European and all other civilizations; for whereas the influence of soil is limited and stationary, the development of man's resources, as favoured by a mild and temperate climate, is probably unlimited and ever increasing. Hence the stationary character of Asiatic civilizations; hence the constantly progressive character of those of Europe.

Besides the accumulation of wealth, a most important question arises as to its distribution, which in advanced societies depends upon very complex laws, but at an early stage seems to be governed in great part, if not entirely, by "physical laws; these laws are, moreover, so active as to have invariably kept a vast majority of the inhabitants of the fairest portion of the globe in a condition of constant and inextricable poverty." We must pass over this part of the subject very briefly, omitting all arguments connected with interest, profit, wages, and rent. After the first tendency towards a division of society into the two classes of workers and non-workers, the increase of the latter will be determined in great part by the presence of a cheap and plentiful national food. In consequence of this increase, the labour-market will be overstocked, wages will be low, and a great amount of poverty will be the result. Such is Mr. Buckle's theory; to discuss it would lead us too far from our special points of interest, and plunge us into the inextricable mazes of political economy.

The fact of the inhabitants of cold climates requiring a very carbonized form of food, whilst those of warm latitudes require one more oxydized, has a powerful influence upon civilization; for whereas the latter kind of food is produced by the earth abundantly and almost without labour, the former, being chiefly of animal origin, is more difficult to obtain. The result is, that in these colder climates a more adventurous and bolder spirit has generally been observed, even in the infancy of society, than in the inhabitants of warmer countries. Tropical populations, as a rule, increase rapidly; therefore wages are low, and poverty prevalent; whilst in cold climates the increase is less rapid, the labour-market is not overstocked, and wages are higher. Hence it arises that in temperate climates there is a very much more equable distribution of wealth, a consequent development of a middle class and of public opinion, and as the natural result, a greater capability of permanent and progressive civilization. One exception to this distinction between European and Asiatic populations is found in Ireland,—the only country in Europe, according to Mr. Buckle, which possesses a very cheap national food, the potato. The population of Ireland, before the aspect of the country was changed by pestilence and emigration, was increasing at the rate of 3 per cent., whilst that of England was only increasing $1\frac{1}{2}$ per cent. Mr. Inglis calculates the average rate of Irish wage at fourpence per day.

To the extremely unequal diffusion of wealth, its concentration amongst the higher classes, and the abject poverty of the lower, is ascribable the transitory and imperfect nature of the Asiatic civilizations. In India, for instance, where on this view the abundance and cheapness of the national food—rice—is the source of the evil, the upper classes are enormously rich, the lower miserably poor. The difference in wealth is attended by a corresponding inequality in social and political power.

"It is not therefore surprising, that from the earliest period to which our knowledge of India extends, an immense majority of the people, pinched by the most galling poverty, and just living from hand to mouth, should always have remained in a state of stupid debasement, broken by incessant misfortune, crouching before their superiors in abject submission, and only fit either to be slaves themselves, or to be led to battle to make slaves of others."—(p. 66.)

The miserable condition of the lowest Hindus or Sudras is something almost inconceivable to us. If one of them speaks of a Brahmin contemptuously, his mouth is burned; if he insult him, his tongue is slit; if he injures him, he is put to death. If he listen to the reading of the sacred books, boiling oil is poured into his ears; if he commit them to memory, he is killed. He is forbidden by law to accumulate wealth, and continues a slave in estimation although freedom be given him; "*for* (says the lawgiver), *of a state which is natural to him, by whom can he be divested?*"

"In India, slavery—abject, eternal slavery—was the natural state of the great body of the people; it was the state to which they were doomed by physical laws impossible to resist. The energy of those laws is, in truth, so invincible, that wherever they have come into play they have kept the productive classes in perpetual subjection. There is no instance on record of any tropical country, in which wealth having been extensively accumulated, the people have escaped their fate; no instance in which the heat of the climate has not caused an abundance of food, and the abundance of food caused an unequal distribution, first of wealth, and then of political and social power. Among nations subjected to these conditions, the people have counted for nothing;—thus have been generated habits of tame and servile submission, by which they have always been characterized. For it is an undoubted fact that their annals furnish no instance of their having turned upon their rulers, no war of classes, no popular insurrections, not even one great popular conspiracy."—(p. 73.)

It would appear as though this passage must have been written before the recent events in India. We give it, however, as an illustration of the general views of our author.

What rice was to India, that was the date to Africa, and particularly Egypt. The palm-tree supplies millions with their daily food in Arabia, and almost the whole of Africa north of the Equator. In one part of Upper Egypt, the Said or Thebaid, there is a food even more liberal in its returns to cultivation than the date—*i.e.*, the dhourra, which yields 240 to 1 in produce. The lotus, also, and a profusion of plants and herbs growing spontaneously, afforded food to the Egyptians. Thus, in Egypt the people multiplied very rapidly, because at the same time that the earth was so prolific, the nature of the climate was such that the inhabitants only required small quantities of food; and as the result, Egypt was probably more thickly populated than any country of the ancient world. As in India, the wealth was very unequally distributed, and the result was the same—a nation of tyrants and slaves. This is shown partly by the stupendous and costly buildings which still remain; for "no wealth, however great, no expenditure, however lavish, could meet the expense which would have been incurred if they had been the work of free men, who received for their labour a fair and honest reward. The people at large were little better than beasts of burden; and all that was expected from them was an unremitting and unrequited labour." The reckless prodigality of labour may be conceived, when we know that 2000 men were occupied three years in carrying one stone from Elephantine to Sais; that one canal cost the lives of 120,000 men; and that 360,000 men were engaged for twenty years building one of the pyramids. Instead, then, of viewing these mighty works as evidences of a high state of civilization, we must consider them as signs of a condition almost utterly depraved and barbarous.

In America, the same laws have been found to prevail. The earliest civilizations were located in only those parts which were at once hot and moist—two conditions causing abundant fertility, and consequent plenty. What rice was to India, and the date to Egypt, that was maize to the inhabitants of Mexico and Peru. The returns of this plant are enormous, averaging in New California seventy or eighty-fold, and in Mexico Proper, three, four, and even eight hundred-fold. The same results occurred here as in India and Egypt—the upper classes were tyrants; the lower, slaves; and there was little or no medium. The arts were cultivated with some success, but there was no power of diffusion in the scanty civilization which they possessed; there was no such thing as public opinion, but a stern despotism of the higher classes, and a slavish subserviency of the lower, who were separated from the former by the impassable barriers of *caste* and legislation. The expenditure of labour was as lavish as in Egypt: the erection of the Royal Palace of Peru occupied 20,000 men for fifty years; and that of Mexico cost the labour of 200,000 men.

The natural advantages of heat and moisture may, however, be so much in excess as to prevent any form of civilization whatever. Such is the case in Brazil, which, from its situation and natural advantages, might be expected to have been the seat of such civilization as similar physical causes had produced in other parts of the world. But the moisture here is in excess; the trade wind pours deluges of rain upon the country, producing the most destructive torrents at certain times. This abundant supply of water, aided by the vast river system and the heat of the climate, stimulates the earth into a productive activity unequalled in any other part of the world. In his description of Brazil, Mr. Buckle evinces descriptive powers of no ordinary kind. We make no apology for quoting at length:—

"Brazil, which is nearly as large as the whole of Europe, is covered with a vegetation of incredible profusion. Indeed, so rank and luxuriant is the growth, that nature seems to riot in the very wantonness of power. A great part of this immense country is filled with dense and tangled forests, whose noble trees, blossoming in unrivalled beauty, and exquisite with a thousand hues, throw out their produce in endless prodigality. On their summit are perched birds of gorgeous plumage, which nestle in their dark and lofty recesses. Below, their base and trunks are crowded with brushwood, creeping plants, innumerable parasites, all swarming with life. There, too, are myriads of insects of every variety; reptiles of strange and singular form; serpents and lizards spotted with deadly beauty; all of which find means of existence in this vast workshop and repository of nature. And that nothing may be wanting to this land of marvels, the forests are skirted with enormous meadows, which, reeking with heat and moisture, supply nourishment to countless herds of wild cattle, that browse and fatten on their herbage; while the adjoining plains, rich in another form of life, are the chosen abode of the subtlest and most ferocious animals, which prey on each other, but which, it might almost seem, no human power can hope to extirpate. Such is the flow and abundance of life by which Brazil is marked above all the other countries of the earth.

"But amid this pomp and splendour of nature, no place is left for man; he is reduced to insignificance by the majesty with which he is surrounded; the forces that oppose him are so formidable, that he has never been able to make head against them—never able to rally against their accumulated pressure. The whole of Brazil, notwithstanding its immense apparent advantages, has always remained entirely uncivilized—its inhabitants wandering savages, incompetent to resist those obstacles which the very bounty of nature has put in their way. . . . . The progress of agriculture is stopped by impassable forests, and the harvests are destroyed by innumerable insects. The mountains are too high to scale, the rivers are too wide to bridge; everything is contrived to keep back the human mind, and repress its rising ambition. It is thus that the energies of nature have hampered the spirit of man."—(pp. 94-6.)

The whole of the immense territory of Brazil, more than twelve times as large as France, contains only about six millions of inhabitants; and these are in the same state of barbarity as they ever were, no sign of progress or civilization being manifest.

Such are the relations in which, according to Mr. Buckle's view, the two elements, soil and climate, bear to civilization, more particularly in the aspect of the accumulation and distribution of wealth. And in precisely the same relation to the accumulation and distribution of thought does he conceive the aspects of nature to stand:—

"It now remains (says he) for me to examine the effect of those other physical agents to which I have given the collective name of Aspects of Nature, and which will be found suggestive of some very wide and comprehensive inquiries into the influence exerted by the external world in predisposing men to certain habits of thought, and thus giving a particular tone to religion, arts, literature, and, in a word, to all the principal manifestations of the human mind."

We shall endeavour to give a succinct account of our author's views on this point; only premising that in this, as in the subjects just discussed, he falls into the error of mistaking those circumstances which *favour* any particular development, mental or physical, for the *efficient and prime cause* of such development. This error is natural and inevitable in any mind which places its trust in nature and man alone, recognising no overruling agency, nor, in short, any principle whatever save *natural progress*.

The aspects of nature influence the mind in a twofold manner—through the imagination and through the reason. With regard to natural phenomena, whatever inspires terror or wonder, and excites ideas of the vague and uncontrollable, has a tendency to stimulate the imagination, and to dominate over the reasoning faculties. Man then feels his inferiority to nature; and "his mind, appalled by the indefined and indefinable, hardly cares to scrutinize the details of which such imposing grandeur consists." On the other hand, when the phenomena of nature are less imposing, and the physical aspects of a country more under the control of man, the imagination is less excited; man becomes able to experiment upon the powers of nature—to bring them in some sort into subjection to him; and the result is, that *reason* asserts her sway. It is thus that the earlier civilizations, being all seated near the tropics, where the aspects of nature are most sublime and terrible, were marked by much superstition, and a great development of the imaginative faculties. Earthquakes are amongst the most fearful of the cosmical phenomena, and "there can be no doubt as to the effect they produce in encouraging particular associations and habits of thought." The mind becomes anxious and timid, and oppressed with a sense of its own helplessness. "Human power failing, superhuman power is called in; the mysterious and the invisible are believed to be present; and there grow up among the people those feelings of awe and of helplessness on which all superstition is based, and without which no superstition can exist." Wherever these and analogous phenomena exist, the imagination dominates over the reason, and produces its natural effects upon the psychical manifestations in the form of superstition, and in the development of the fine arts rather than of the sciences. Thus Spain, Portugal, and Italy—the countries of Europe where earthquakes are most frequent—have produced most of the great painters and sculptors of Europe. The purely reasoning faculties are undeveloped, and it is here that superstition has taken its deepest hold of the people. In some other countries, all serious dangers are viewed as supernatural occurrences, and are not only submitted to, but worshipped; as is the case amongst some of the Hindus.* Some, from feelings of reverential fear, refuse to destroy wild beasts and noxious animals.

"Summing up these facts, it may be stated that, in the civilizations exterior to Europe, all nature conspired to increase the authority of the imaginative faculties, and weaken the authority of the reasoning ones. . . . . In Europe this law is opposed by another diametrically opposite, by virtue of which the tendency of natural phenomena is, on the whole, to limit the imagination and embolden the understanding;

* The Hindus in the Iruari forests (says Mr. Edye) worship and respect everything from which they apprehend danger.

thus inspiring man with confidence in his own resources, and facilitating the increase of his knowledge, by encouraging that bold, inquisitive spirit which is constantly advancing, and on whic hall future progress must depend."—(pp. 118-9.)

The civilization of Europe has diverged from all those that preceded it; and this is supposed to be owing to the operation of the causes alluded to, since—1. There are certain natural phenomena which act on the human mind by exciting the imagination; and 2. These phenomena are much more numerous out of Europe than in it. The mode of operation of these phenomena is illustrated by their effects upon the literature, religion, and art of India and Greece respectively, "these being the two countries respecting which the materials are most ample, and in which the physical contrasts are most striking."

The ancient literature of India is marked by the ascendancy of an imagination luxuriant even to disease. Almost the whole of their writings are poetical: their works on grammar, law, history, medicine, mathematics, geography, metaphysics, are all in a regular system of versification. Their geographical and chronological systems, apparently the least likely to be so affected, are full of imaginative flights, connected with an exaggerated respect for past ages, and a belief in the extraordinary longevity of ancient times.* The Institutes of Menu also are said to have been revealed to man about two thousand million years before the present era.

"Not only in literature, but also in religion and art, this tendency (to exaggeration) is supreme. To subjugate the understanding and exalt the imagination is the universal principle. In the dogmas of their theology, in the character of their gods, and even in the forms of their temples, we see how the sublime and threatening aspects of the external world have filled the minds of the people with those images of the grand and terrible, which they strive to reproduce in a visible form, and to which they owe the leading peculiarities of their national culture."

* The common duration of life was 80,000 years; holy men lived 100,000 years; some of the poets lived to the age of half a million. But all these are short lives compared to that of "a very shining character in Indian history, who united in his single person the functions of a king and a saint. This eminent man lived in a pure and virtuous age, and his days were indeed long in the land, since when he was made king he was 2,000,000 years old: he then reigned 6,300,000 years; having done which, he resigned his empire, and lingered on for 100,000 years more."—*Asiatic Researches.*

Mr. Buckle on this subject takes the opportunity of classifying the Hebrew and Christian writings with these fables. "A belief in the longevity of the human race, at an early period of the world, was the natural product of those feelings which ascribed to the ancients an universal superiority over the moderns; and this we see exemplified in some of the Christian, and in many of the Hebrew writings. But the statements in these works are tame and insignificant when compared with what is preserved in the literature of India."—(pp. 122-3.)

Whilst such are the effects of the wondrous and fearful works of nature in India, in Greece the aspects of nature are so different, that "the very conditions of existence are changed." The country is of small extent, easy of access from others; the mountains are less, the streams are smaller; earthquakes are less frequent, the climate more healthy, hurricanes less disastrous, wild beasts and noxious animals less abundant. Thus, whilst in India the tendency of natural phenomena was to inspire fear, in Greece it was to give confidence.

"In Greece, therefore, the human mind was less appalled and less superstitious; natural causes began to be studied; physical science first became possible; and man, gradually waking to a sense of his own power, sought to investigate events with a boldness not to be expected in those other countries, where the pressure of nature troubled his independence, and suggested ideas with which knowledge is incompatible."—(p. 127.)

Whilst the deities of both India and Greece retain somewhat of human attributes, in the former they are all exaggerated into images of the extremest terror,* whilst in the latter they were all strictly human; stronger, or more beautiful than man they might be, but still never grotesque. The actions of the Indian gods were all preternatural; those of Greece had human tastes, pursuits, and passions, and most of them were merely embodiments of the emotional ideas. In Greece, also, we first meet with hero-worship, the approximation of humanity to deity. "In Greece, for the first time in the history of the world, the imagination was in some degree tempered and confined by the understanding. . . . . Whether or not the balance was accurately adjusted, is another question; but it is certain that the adjustment was more nearly arrived at in Greece than in any previous civilization."

We cannot pause to trace the resemblance between the evolutions of these laws in the other ancient civilizations; but having arrived at the verge of the permanent forms of civilization, we must now follow our author in his investigation of the influence of mental laws upon the progress of society, under the twofold aspect of *moral* and *intellectual* development. Mr. Buckle in the outset recognises this division, "for" (says he) "there can be no doubt that a people are not really advancing, if, on the one hand, their increasing ability is accompanied by increasing vice; or if, on the other hand, while they are becoming more virtuous, they

* Siva is a hideous being, with a girdle of snakes, a human skull in his hand, and with a necklace of human bones. He has three eyes, is clothed in a tiger's skin, and over his left shoulder the deadly cobra di capello rears its head. His wife, Doorga, is still more revolting in appearance, and has an insatiable appetite for blood.

likewise become more ignorant." Acknowledging, then, the necessity for the mutual working together of these two elements, morality and intellect, Mr. Buckle proceeds to inquire which of the two is the "more important." Guarding at first against the interpretation of "natural progress" into a progress of natural capacity, which may or may not be the case, and at all events is not proved, he shows that this progress is one of *opportunity*—of external advantage, not of internal power. It is here that Mr. Buckle's most dangerous subtleties commence. On this seemingly innocent basis he founds an argument, feeble yet plausible, abounding in sophistry, by which he attempts to prove the very small influence which morals exert upon the progress of societies. The line of reasoning is to this effect:—The moral and intellectual conduct of the aggregate of men is regulated by the moral and intellectual notions prevalent in their time. Some will go beyond, some will fall short of this standard; but, generally, this will be the rule. Now, this standard is continually varying; the paradox or heresy of one period is the received doctrine of the next: a mutability which shows that "the conditions on which the standard depends must be themselves very mutable; and these conditions, whatever they may be, are evidently the originators of the moral and intellectual conduct of the great average of mankind." Knowing, then, that the main cause of human actions is necessarily extremely variable, if any set of circumstances claim to be such cause, we have but to apply the test of variability, in order to adjudicate on their claims. The next step in the argument is so daring in its unscrupulous assertions, that we give it at length in the writer's words:—

"Applying this test to moral motives, or to the dictates of what is called moral instinct, we shall at once see how extremely small is the influence those motives have exerted over the progress of civilization. For there is, unquestionably, nothing to be found in the world which has undergone so little change as those great dogmas of which moral systems are composed. To do good to others; to sacrifice for their benefit your own wishes; to love your neighbour as yourself; to forgive your enemies; to restrain your passions; to honour your parents; to respect those who are set over you—these and a few others are the sole essentials of morals; but they have been known for thousands of years, and not one jot or tittle has been added to them by all the sermons, homilies, and text-books which moralists and theologians have been able to produce."—(p. 163.)

It is added, in a note to this passage:—

"That the system of morals propounded in the New Testament contained no maxim which had not been previously enunciated, and that some of the most beautiful passages in the Apostolic writings are quotations from Pagan authors, is well known to every scholar; . . . and to assert that Christianity communicated to man moral truths previously unknown, argues, on the part of the assertor, either gross ignorance or wilful fraud."

And, as a quotation from Sir J. Mackintosh:—

"More than 3000 years have elapsed since the composition of the Pentateuch; and let any man tell me, if he is able, in what important respect the rule of life has varied since that distant period. The fact is evident, that no improvements have been made in practical morality."

This assumed stationary character of morality is contrasted forcibly with the advance of the intellectual sciences; and then comes the conclusion, that as civilization is the product of moral and intellectual agencies, and "since that product is constantly changing, it evidently cannot be regulated by the stationary agent,"—and, therefore, the only other agent being the intellectual one, this *must be and is* the real mover of all civilization. The good or evil works of men or states die with them (!), but the intellectual contributions are preserved, and become "the heirlooms of mankind—the immortal bequest of the genius to which they owe their birth." These remarkable views are modestly summed up as follows:—

"These conclusions are, no doubt, very unpalatable; and what makes them peculiarly offensive is, that it is IMPOSSIBLE TO REFUTE THEM. For the deeper we penetrate into this question, the more clearly shall we see the superiority of intellectual acquisitions over moral feelings." —(p. 166.)

Although these arguments are such that Mr. Buckle is "quite unable to see on what possible ground their accuracy is to be impugned," we must confess them to be far otherwise than convincing. We hesitate which most to admire in all this—the arbitrary manner in which Mr. Buckle excludes the Maker from all participation in the conduct of the affairs of His creatures; the gratuitous assumption that his classification of the sources of human action is and must be an exhaustive one; the feeble inefficiency of method in the argument built upon so sandy a foundation; the self-complacency with which the conclusions are deemed irrefragable; or the threefold crowning error, in *fact*, in *inference*, and in *philosophy*, with which the ingenious structure is completed. We are indebted to him, however, for *one* classification which we *do* accept as exhaustive—viz., the division of the motives for misrepresentation into "*gross ignorance* or wilful fraud;" we accept it, and proceed to show that he himself is amenable to one or other of the allegations.

Firstly, in reference to the error as to *fact* with which we charge the author.

When Mr. Buckle alleges that the laws of morality have always remained the same, or have been unaltered for thousands of years, since the composition of the Pentateuch, and that Christianity revealed nothing new, can he possibly be ignorant that the Great Author of our faith, in his personal ministry, changed the entire spirit of the Mosaic law? Speaking to a people who were jealous of the slightest misinterpretation of their law, and who were intently watching for some grounds for accusation against him, he stated that the social spirit of their Levitical code was "an eye for an eye, and a tooth for a tooth." And who had ever heard in the Heathen world of such a law as that which was then and there substituted for this—"*Resist not evil;* LOVE *your enemies;* BLESS *them that curse you;* PRAY *for them which persecute you?*" *Is* Mr. Buckle "grossly ignorant" of this, or is his statement a "wilful fraud"?

So much as to the error in fact, of which many other illustrations might be given; but this is sufficient to show the loose, careless manner in which our author treats all matters which do not tend directly to the support of his foregone conclusions.

But, even supposing, for the sake of argument, that the moral law was a constant quantity; is its PROGRESSIVE RECOGNITION, and its *reception* as a rule of life, to count for nothing in man's progress? Because constant, is it inoperative, where, by means of teaching from without, or more energetic inculcation within, it is more forcibly impressed upon a community? Apply the same hypothesis to intellectual attainments: suppose that the entire present knowledge of European science could be transfused at once into China or some other Asiatic nation, the absolute grade of human knowledge would not be advanced one step, yet the nation would be greatly advanced in civilization. The laws of nature have been the same from the foundation of the world; it is only the ever-increasing *recognition* of them which has produced intellectual development—only their *application* to art and science which has led to the advance in them. And so, although the moral law may have been constant for two thousand years, it is to its *recognition* that we look for the progress of humanity on a fixed basis.

The third error—that in philosophy—is connected with the assumed superiority of the intellect over the moral nature, because the one is *cumulative* in its results, and the other not so. The *fact* is doubtless so in one point of view—we do not recognise moral truth more readily nor clearly because others have recognised it before us; whilst intellectual truths, being constantly appropriated and made subservient to the uses of man, to his physical, moral, and spiritual desires, become permanent in the form of the machinery of society. But not on this account are the intellectual laws to be esteemed superior to the moral.

"It is exactly in proportion as intellectual power is capable of yielding fruits which are *non*-intellectual, that it is more cumulative than moral or spiritual power. In other words, so far as the intellect can be made the effective instrument of other human desires and capacities beside the intellect, so far is it more cumulative than faculties which have no end out of themselves But this is only saying that intellectual agencies are subsidiary and instrumental to moral and spiritual agencies, while the latter are not subsidiary and instrumental to the former. . . . . The intellectual laws are, in fact, immediately subordinated to the physical, moral, and spiritual desires. . . . . The intellect is cumulative in Mr. Buckle's sense, only because its results are fitly instrumental to desires that are other than intellectual; while the *higher capacities* of human nature have their fittest ends only in themselves, and are utterly distorted and defaced by being made the instruments of lower capacities."*

Mr. Buckle illustrates his principles by the decline of what he considers the two greatest evils that have ever afflicted humanity —viz., religious persecution and war. As to the former, he considers that he has proved that it is essentially an intellectual process, and that no good could be effected by the operation of moral feelings.

"The causes of the decline of the warlike spirit . . . is owing to the increase of the intellectual classes, to whom the military classes are necessarily antagonistic. In pushing the inquiry a little deeper, we have, by still further analysis, ascertained the existence of three vast though subsidiary causes, by which the general movement has been accelerated. These are, the invention of gunpowder, the discoveries of political economy, and the discovery of improved means of locomotion. . . . We are bound to infer that the two oldest, greatest, most inveterate and most widely-spread evils which have ever been known, are constantly, though, on the whole, slowly diminishing; and that their diminution has been effected not at all by moral feelings nor by moral teachings, but solely by the activity of the human intellect, and by the inventions and discoveries which, in a long course of successive ages, man has been able to make."

Mr. Buckle also pledges himself to show, in his future volumes, that the progress which Europe has made from barbarity to civilization is "entirely due to its intellectual activity," and that

* "National Review," January, 1858. We owe an apology to the able writer of the Article on "Civilization and Faith," for appropriating his expressions on this subject.

"the changes in every civilized people are, in their aggregate, dependent solely on three things—first, on the amount of knowledge possessed by their ablest men; secondly, on the direction which that knowledge takes—that is to say, the sort of subjects to which it refers; thirdly, and above all, on the extent to which the knowledge is diffused, and the freedom with which it pervades all classes of society." —(p. 205.)

Mr. Buckle is too close an observer to be able to ignore the conviction that the actions of *individuals* are greatly affected by their moral feelings and their passions; but, inasmuch as these are antagonistic to the passions of others, their effects are neutralized, and "the total actions of mankind, considered as a whole, are left to be regulated by the total knowledge of which mankind is possessed." With that strange one-sidedness of argument which characterizes the whole work, it never seems to occur to him that knowledge or intellectual opinion may in its divisions neutralize itself, or be neutralized by ignorance; and that a determined opponent might with equal reason assert that the knowledge and ignorance of mankind being continually at war, the total actions of man were left to be regulated by such moral sentiments as they possess. But we reserve our general comments on these views until we have examined the author's opinions on the three great subjects—Religion, Literature, and Government—in their effects upon civilization. These are allowed to be subjects of vast importance,

"and which (says Mr. Buckle), in the opinion of many persons, are the prime movers in human affairs. That this opinion is altogether erroneous, will be amply proved in the present work; but as the opinion is widely spread and is very plausible, it is necessary that we should come at once to some understanding respecting it, and inquire into the real nature of that influence which these three great powers do actually exercise over the progress of civilization."

It might be amusing, were the subject less serious, to observe the extremely off-hand manner in which Mr. Buckle disposes of the most important considerations connected with the progress of society. A country which holds no communion with other countries, but is completely isolated, will, or rather *would*, of necessity, form its own religion (premising the absence of a revealed one), its literature, and its government; and these would evidently be the results and symptoms of the state of society, rather than its cause. But we have already shown that one of the most important agencies influencing any given civilization, is the intercourse of the country with others in a higher state of cultivation. It will scarcely be credited, unless we quote the writer's own words, that he briefly sums up this powerful agency under the head of "*tampering*" with natural progress; considers the religion, literature, and government of nations merely as symptoms; and attributes no influence, save a deleterious one, to any efforts made from without to assist in the amelioration of a debased condition.

"Out of a certain condition of society, certain results naturally follow. Those results may, no doubt, be tampered with by some external agency; but if that is not done, it is impossible that a highly-civilized people, accustomed to reason and to doubt, should ever embrace a religion of which the glaring absurdities set reason and doubt at defiance." . . . . "The truth is, that the religious opinions which prevail in any period are among the symptoms by which that period is marked. When the opinions are deeply rooted, they do, no doubt, influence the conduct of men; but before they can be deeply rooted, some intellectual change must have taken place. We may as well expect that the seed should quicken in the barren rock, as that a mild and philosophic religion should be established among ignorant and ferocious savages."—(p. 233.)

It naturally follows, that the author throws all possible discredit upon missionary enterprise and narrative, inasmuch as a broad denial of the accuracy of these statements is quite necessary for the support of his doctrine. Yet, accustomed as we had become to his habit of dogmatism and rash assertion, we were somewhat startled on meeting with the following passage:—

"After a careful study of the history and condition of barbarous nations, I do most confidently assert that there is no well-attested case of any people being permanently converted to Christianity, except in those very few instances where missionaries, being men of knowledge as well as men of piety, have familiarized the savage with habits of thought, and, by thus stimulating his intellect, have prepared him for the reception of those religious principles which, without such stimulus, he could never have understood."—(p. 234.)

And this is written by an inhabitant of England, a country of all others affording the most remarkable refutation of such an assertion! For what were the people of England but idolatrous barbarians before St. Augustine's mission? And we hear nothing of his having familiarized the savages of our island with habits of thought before he preached to them the pure Gospel of the kingdom. Let us hear what a recent writer, an enemy to missions, if not to Christianity itself, says:—

"Beginning with the early times, however, we are first struck with the thought of what we ourselves owe to missionary enterprise. In the south of England and in Ireland, there was probably some early preparation, by the influx of persecuted Christians from the Continent; but the great release from the iron rule of Druid caste-tyranny we owe to St. Augustine and other missionaries, who came for the express purpose of making us Christians."

It may be asserted that we were not savages; but whether we consider the religion of a nation in the light of *cause* or *symptom* of its condition, we may feel certain that the Druid faith can only coexist with a very low development of intellect, and is incompatible with any high form of civilization. Mr. Buckle gives some illustrations of his views of the introduction of a religion too pure for the intellectual state of the people, which appear to us much more rash and bigoted than many of the opinions of which he complains, and irreverent in the extreme from their utter disregard of all those principles which are held sacred in a Christian land. He presupposes that it is a mark of the utmost intolerance to blame a person for irreverence, because he does not revere what we do. But he who, in a Christian country, takes every opportunity of sneering at the faith and creed of that country, must expect to meet with the natural return. The doctrine of "One God (says he), taught to the Hebrews of old, remained for many centuries altogether inoperative. The people to whom it was addressed had not yet emerged from barbarism; they were, therefore, unable to raise their mind to so elevated a conception." In other words, the revealer of that religion committed a great mistake. Because of the lapses into idolatry which so often characterized this people, Mr. Buckle can quietly overlook the potency of those influences which for three thousand years have kept the Jews a "peculiar people," separate from all the other nations. In like manner, the introduction of the Christian religion was altogether an anachronism and a mistake:—

"The Romans were, with rare exceptions, an ignorant and barbarous race; ferocious, dissolute, and cruel. For such a people, Polytheism was the natural creed; and we read, accordingly, that they practised an idolatry which a few great thinkers, and only a few, ventured to despise. The Christian religion falling among these men, found them unable to appreciate its sublime and admirable doctrines. And when, a little later, Europe was overrun with fresh immigrations, the invaders, who were even more barbarous than the Romans, brought with them those superstitions which were suited to their actual condition. It was upon the materials arising from these two sources that Christianity was now called to do her work. The result is most remarkable; for after the new religion seemed to have carried all before it, and had received the homage of the best part of Europe, it was soon found that nothing had been really effected."

It will be observed that we have not often interrupted the course of this analysis to give *historical* refutations of the glaring errors into which our author, by viewing casualties as essentials, and essentials as casualties, is perpetually falling. But here we must examine the question in a little more detail. Perhaps from Mr. Buckle's point of view, considering all religion as entirely secondary and subordinate to intellectual development, and all morality as nearly inoperative in society, it may seem that *nothing was done* when a pure form of religion (with certain growing corruptions) was introduced in the place of one which permitted all society to be one mass of corruption. "The Roman empire included," says Shaftesbury, "a race of men who seemed to vie with each other in the commission of as grand crimes, and in the perpetration of as odious vices, as ever disgraced humanity." Vice was national, sanctioned by the highest examples, followed by the lowest of the people, soaking through every grade of society. The satirists of those days present a picture of society unfit to translate, almost even to allude to. "Murder, and every variety of unutterable crime characterized that declining age; and had not the Almighty mercifully interposed, the human race ran the risk of being extinguished by the pressure of its own detestable vices."—*Fraser*. That the introduction of Christianity did not do all that might have been hoped in a better state of society, is matter of history; but to say that "nothing was done," argues either "gross ignorance or wilful fraud," in the face of the edicts of the Emperor Constantine alone, the practical benefits of which were immediate and visible. For, says the writer just quoted, "the laws concerning slavery were remodelled and mitigated, abduction and adultery were visited with severe punishments, divorce was subjected to intelligible restrictions, and some of the more obvious vices of the age were removed by the improved tone of public opinion."

We cannot enter more deeply into the question why Christianity failed to regenerate man completely; to do so would require volumes, instead of pages; but we notice this one point to show how Mr. Buckle slurs over all history which is not in accordance with his theory of development, and then proves from this *historia expurgata* that his theory must be the true one. Let us, however, before leaving the subject, retort upon him by asking, if Christianity did so little, what was the much-boasted "natural progress" doing? What of the intellectual activity, to the effects of which Mr. Buckle pledges himself to trace *all the progress* that Europe has made in civilization?* Why did the gorgeous civilizations of Greece and Rome decay? Was it that the intellectual activity of the Golden Age was less than in the

* See p. 204.

days of the commonwealth of Rome? Was it that the age of Socrates, of Plato, of Pythagoras, Pindar, Æschylus, Sophocles, Euripides, of Herodotus and Xenophon, was an age of mental stagnation and decay? Were not these times when the thinking faculties of man were developed to an extent since unknown—when thought received an impulse, and a direction, and a form which have never ceased to operate upon it? In one at least of these empires, also, there was, in addition to its intellectual activity, a powerful middle class, and a "public opinion." Yet the empire fell, and upon its ruins wandering hordes of barbarians fixed their seat. Why was this? In answering the question, we will strictly follow Mr. Buckle's method of induction.*

If the causes of progress in civilization are moral and intellectual, the causes of decay are necessarily of the same order, and depend upon a deficiency of one or other element. But as we see that at the times of the decay of the Greek and Roman empires the intellectual element was in full vigour, we are compelled to attribute the phenomenon to the absence of the moral element—a theory which will be amply supported by the evidence of the times themselves; for, "among the people the most unblushing and disgusting profligacy was common, with all the immorality and all the vices that can disgrace human nature. To this general corruption of manners may be added, levity of character, a total disregard of decency, laxity of social relations, and grossness of political institutions. Such were the causes of the downfall"† of these empires. Thus, by his own method, our author is confuted; and, like him, we are "quite unable to imagine on what possible grounds" the force of this view can be contested.

Religion, literature, and government are classed by Mr. Buckle as "disturbing causes"‡ in civilization. In reference to the second, the same views are promulgated as those on the first.

* "Since civilization is the product of moral and intellectual agencies, and since that product is constantly changing, it evidently cannot be regulated by the stationary agent; because, when surrounding circumstances are unchanged, a stationary agent can only produce a stationary effect. The only other agent is the intellectual one; and that this is the real mover, may be proved in two distinct ways—first, because being, as we have already seen, either moral or intellectual, and being, as we have also seen; not moral, it must be intellectual; and secondly, because the intellectual principle has an activity and a capacity for adaptation, which, as I undertake to show, is quite sufficient to account for the extraordinary progress that, during several centuries, Europe has continued to make."—(p. 165.)

"By each successive analysis, the field of the inquiry has been narrowed, until we have found reason to believe that the growth of European civilization is SOLELY DUE TO THE PROGRESS OF KNOWLEDGE, and that the progress of knowledge depends on the number of truths which the human intellect discovers, and on the extent to which they are diffused."—(p. 265.)

† Mackinnon's "History of Civilization," vol. i. p. 60.

Literature is "simply the form in which the knowledge of a country is registered." It is a symptom of the state of the people, not a cause of it. It is scarcely credible that the author seems to take no account of the influence of literature from without; nor does he dwell more on the effect of great lights within than this passing notice:—

"Individual men may of course take great steps, and rise to a great height above the level of their age. But if they rise beyond a certain point, their present usefulness is impaired; if they rise still higher, it is destroyed."

The author must have felt sensible of the feebleness of this part of his subject, when he wrote the following profound observations:—

"The truth is, that although Europe has received great benefit from its literature, this is owing not to what the literature has originated, but what it has preserved"! . . . . "Knowledge must be acquired before it can be written"!! . . . . "Literature, in itself, is but a trifling thing, and is merely valuable as being the armoury in which the weapons of the human mind are laid up; and from which, when required, they can be drawn"!!!

The same principles are applied to the third "disturbing cause," government, which is, or ought to be (in Mr. Buckle's view), nothing more than an exponent of popular progress. When it has attempted to be anything more than this, all "that it has done has been done amiss."

"The other opinion to which I have alluded is, that the civilization of Europe is chiefly owing to the ability which has been displayed by the different governments, and to the sagacity with which the evils of society have been palliated by legislative remedies. To any one who has studied history in its original sources, this notion must appear so extravagant as to make it difficult to refute it with becoming gravity. Indeed, of all the social theories which have ever been broached, there is none so utterly untenable, and so unsound in all its parts, as this."—(p. 250.)

A sufficient proof of this is supposed to be afforded by the repeal of the Corn Laws, and the Reform Bill, which, being in their origin popular movements, carried Government along with them. The whole system of taxation is repudiated, as utterly evil; the "laws in favour of industry have injured industry; the laws in favour of religion have increased hypocrisy; and the laws to secure truth have encouraged perjury;" and one main condition of the prosperity of a people is, "that its rulers shall have very little power, that they shall exercise that power very sparingly, and that they shall by no means presume to raise themselves into supreme judges of the national interests, or deem themselves authorized to defeat the wishes of those for whose benefit alone they occupy the post entrusted to them."

In alluding to the history of the Middle Ages, Mr. Buckle finds three things to complain of—the art of writing, the change of religion, and the monopoly of history by a certain class. The art of writing was objectionable, because it enabled men to dispense with those invaluable old ballad-singers and their ballads; the change of religion, because it interrupted and interpolated the old traditions; and the third, "more powerful than all, was, that history became monopolized by a class of men whose professional habits made them quick to believe; and who, moreover, had a direct interest in increasing the general credulity, since it was the basis upon which their own authority was built." These men are, of course, the priests and ministers of religion generally, at whom Mr. Buckle takes every opportunity of sneering. In fact, he speaks of all men who believe in an overruling Providence with the most lofty, yet pitying and wondering contempt. This is exemplified in his remarks on Comines:—

"This eminent politician, a man of the world, and skilled in the arts of life, deliberately asserts that battles are lost, not because the army is ill supplied, nor because the campaign is ill conceived, nor because the general is incompetent, but because the people or their prince are wicked, and Providence seeks to punish them. For," says Comines, 'war is a great mystery, and being used by God as the means of accomplishing His wishes, He gives victory, sometimes to one side, sometimes to the other.' . . . . The last vestige of this once universal opinion is the expression, which is gradually falling into disuse, of 'appealing to the God of Battles.'"

In like manner, in the following chapter we meet with a scoff at all prayer or invocation of Divine assistance:—

. . . "We still see the extraordinary spectacle of prayers offered up in our churches for dry weather or for wet weather; a superstition which to future ages will appear as childish as the feelings of pious awe with which our fathers regarded the presence of a comet, or the approach of an eclipse. We are now acquainted with the laws which determine the movements of comets and eclipses, and . . . have ceased to pray to be preserved from them. But because our researches into the phenomena of rain happen to have been less successful, we resort to the impious (!) contrivance of calling in the aid of the Deity to supply those deficiencies in science which are the result of our own sloth; and we are not ashamed, in our public churches, to prostitute the rites of religion by using them as a cloak to conceal an ignorance we ought frankly to confess."

We are neither able nor disposed to contest these points with

Mr. Buckle; his intellectual creed is not ours; his faith is not our faith, nor his God our God; he who has sought so deeply into history will not be shaken by arguments of ours; he that holds that scepticism* is the great adjunct to civilization, and that expediency,† not truth, is the most to be desired politically, will not be ready to relinquish his principles for a despised and, as he supposes, expiring faith. As Nebuchadnezzar, in the pride of his wealth, looked upon the evidences of his riches, and said, "Is not this great Babylon that I have built by the might of my power?" so Mr. Buckle, in the pride of his inexhaustible stores of learning, seems to look forth upon the conditions of human existence, and say that human intellect is sufficient for all this. It is a common error, whilst life is young and strong within us, to believe in and be satisfied with secondary causes, and take no thought of the One Great Cause. But as the problems of life press upon us, and approach to their final solution, it is well that we discover that there is a want of something more than food, and intellect, and scepticism, and expediency. Happy is he to whom this discovery comes in time, and who finds that, let the nations rage as they will, there is indeed ONE that ruleth over the affairs of men.

It will be pleasant, in conclusion, to review the opinions of some others as to the bearing of morality and religion on civilization, that none may be left with the idea that high intellectual attainments tend, of necessity, to a disbelief in all religion as a means of ameliorating the condition of humanity. We will notice only three, whose names will certainly carry some weight—John Milton, Samuel Johnson, and Montesquieu.

Milton says that—

"A commonwealth ought to be but as one huge Christian personage, one mighty growth and stature of an honest man, as big and compact in virtue as in body; for look what the grounds and causes are of single happiness to one man, the same ye shall find them to a whole state, as Aristotle, both in his Ethics and Politics, from the principles of reason, lays down. By consequence, therefore, that which is good and agreeable to the true welfare of every Christian, and that which can be justly proved hurtful and offensive to every true Christian, will be evinced to be alike good or hurtful to monarchy; for God forbid that we should separate and distinguish the end and good of a monarch from the end and good of a monarchy, or of that from Christianity."—*Reformation in England.*

Dr. Johnson, speaking of calamities incident to a want of religion, says:—

"Discord must inevitably prevail among men who have lost all sense of Divine superintendence, and who have no higher motive of

* Vide p. 308. † Vide p. 416.

action or forbearance than present opinion or present interest. Surely there will come a time when every passion shall be put upon the guard by the dread of general depravity; when he who laughs at wickedness in his companion, *shall start from it in his child;* when the man who fears not for his soul, shall tremble for his possessions; when it will be discovered that religion only can secure the rich from robbery, and the poor from oppression,—can defend the State from treachery, and the Throne from assassination."

Let us, finally, hear the testimony of Montesquieu, than whom no one has thought more deeply upon, or entered more fully into, the philosophy of nations:—

"Chose admirable! La religion Chrétienne qui ne semble avoir d'objet que la félicité de l'autre vie, fait encore notre bonheur dans celle-ci.

"C'est la religion Chrétienne qui malgré la grandeur de l'Empire, et le vice du climat, a empêché le despotisme de s'établir en Ethiopie, et a porté au milieu de l'Afrique les mœurs de l'Europe et ses loix. . . . Nous devons au Christianisme et dans le Gouvernement un certain droit politique, et dans la guerre un certain droit des gens, que la nature-humaine ne sçauroit assez reconnoitre."—*Esprit des Loix.*

Before taking final leave of Mr. Buckle, we will briefly review his theory, so as to present it in one view.

Man's civilization is first originated by—1. The nature of the soil, producing food in more or less abundance; 2. By the nature of the climate, influencing the energy and continuity of man's operations upon the soil—these two combining to originate an accumulation of wealth, without which there can be no leisure for thought, and therefore no civilization; and 3. By the aspects of nature, which are to the accumulation of thought, what soil and climate are to that of wealth.

The tropical civilizations are characterized, in consequence of the abundance and cheapness of food, by broad, impassable intervals between high and low—by tyranny and slavery; and in their mental aspect, by the marks of the ascendancy of physical agencies over man, in the development of the imagination and of superstition.

The European civilizations, on the contrary, are marked by the ascendancy of man over nature in the development of the reasoning faculties, and of free inquiry. Hence arises *intellectual progress,* the source of *all the advance* which Europe has made in civilization. In this, scepticism is a prime agent.

Religion, literature, and government are merely "disturbing causes," of which no account need be taken in respect of general or universal progress. Morals influence every individual, but not nations. Literature is a mere nothing. Government is a clog upon the wheels of progress.

Prayer is a folly or an impiety. Providence is an impossibility. Expediency is the proper end and aim of politics.

Such is the author's physical and mental creed. His bitterest scorn is reserved for Christianity. Truly it has been said—"L'homme pieux et l'Athée parlent toujours de religion; l'un parle de ce qu'il aime, et l'autre de ce qu'il craint."

It would appear almost sufficient for the refutation of this theory to have disentangled it from the enormous mass of learning from which it is evolved, and to have placed it in a clear, consecutive form. But it may be useful as briefly to indicate its errors and their sources.

The first and fundamental error is that of considering those circumstances which are merely *favourable to the development* of civilization, as actual "dynamics:" viewing, for instance, the abundance and cheapness of food as the efficient and final cause of the accumulation and distribution of wealth. For it is clear that, in the most prolific lands, there could be neither accumulation nor distribution of wealth were it not for the passions and desires—in other words, for the moral nature of man. What creates an enormously rich aristocracy and a people of slaves but the selfishness of man, and the absence of love and charity? Physical conditions may *favour*, but cannot create, such a state of things.

Another great and cardinal error is that of considering intellectual progress as the sole source of civilization in Europe. We have partly entered upon this subject before; but we may add one or two observations. It is an error to consider morals "stationary," and therefore unable to produce a "progressive" result. For it is almost too evident to require allusion, that, between the condition of the savage and that of the civilized man, there is quite as great a *moral* as an intellectual difference. And again, experience proves that intellect alone is unable to found a permanent civilization. For why, we again ask, did the civilizations of Greece and Rome decay at a time when intellect was more active than at any previous period? Mr. Buckle responds that the intellect was indeed active, but that there was no power of diffusion in it. True. But what is this but to say that intellect *of itself* has no diffusive power, consequently *of itself* no civilizing power? And upon what does this spirit of diffusion depend, but the presence amongst the intellectual classes of the constantly operative moral law of "loving our neighbour as ourselves?" It is the moral principle of self-negation, and love and charity to our fellow-creatures, that makes the difference between a diffusible and non-diffusible intellect. It is true that this moral law may be considered in effect the same—*i.e.*, stationary in all ages—but only as a theory; in practice it requires perpetual *re*-investigation and perpetual *re*-reception. Its presence or absence in any community makes the difference between a permanent and a transitory civilization; and this is equally in accordance with theory and experience.

But we go further than this, and assert that Mr. Buckle entirely mistakes the philosophy of intellectual development, as bearing upon the progress of nations. For intellect of itself is not necessarily expansive—it is *self-contained;* its improvement is to some extent dependent upon the fruits of past intellect, but its immediate operation is upon individual minds, which do not *necessarily* impart their knowledge; so that intellect *may be,* and *is* in its essence, *unsocial.* But not so with morals, for these have reference expressly to the relational faculties of man, and cannot be exercised *except* relatively to others. These, then, are of constant operation, and enter into and ramify through all societies and communities: whilst the communion of the intellect, however common or frequent it may be, is still not essential, but only voluntary and casual; and the spread of intellectual discoveries *never can* be due to the discoveries themselves, but only to the presence in the discoverer of an impulse arising from some ramification of the moral law, or in obedience to his own desires or passions. Love of fame, love of money, love of mankind—three of the great stimuli to the spread of intellectual discoveries—none of these are in any way intellectual, but have immediate reference to our moral nature.*

We say, then, that what has been proved to be true by experience is equally accordant with theory—viz., that intellect of itself, and disunited from a moral law, is, and must of necessity ever be, utterly inoperative in advancing, consolidating, or pre-

* We have dwelt upon the subject of the influence of the moral law on civilization at great length, for this reason—that it is chiefly to this influence that we look for the regeneration of the "*degenerate*" beings and races that mingle with and corrupt all grades of our civilized societies. Our meaning will be rendered more clear by a reference to the Essay on the "Degeneracy of the Human Race," in this Journal, April, 1857. M. Morel thus speaks of moral treatment :—

"Le traitement moral qui n'est que l'application des devoirs imposés par la loi morale, divine, fixe et immuable, n'est pas une chose nouvelle. La propagation de cette loi, sa pratique, son application aux individus, selon leur age et le degré de leur intelligence, ne sont pas non plus des fonctions exclusivement réservées a quelques hommes, et ne representent pas davantage des devoirs que les uns sont libres d'accepter et les autres de rejeter. . . . . Ceux qui sont chargés de l'appliquer, sont non seulement les moralistes, les prêtres, les magistrats, les instituteurs de la jeunesse, les médecins, mais le père de famille et les membres qui composent la famille.

"Sans doute, la loi morale n'est pas une chose nouvelle, mais l'exposé clair et methodique . . . . de toutes les questions qui ont trait a l'amélioration des masses, autrement dit, a leur *moralisation,* est une science encore toute nouvelle."

Such are the opinions of one of our deepest and most philosophical thinkers, on one of the most difficult problems connected with our social condition and prospects.

serving a civilization. Its inefficiency was proved in all the ancient civilizations, which, whilst intellect was in its most active state, died out for lack of faith, conscience, and a moral law, as a rule of national and private life. In modern times we have had but one instance of a highly-civilized state relapsing (temporarily) into a state of barbarism; and this was synchronous with a preternatural activity of the intellectual faculties, and a formal national rejection of the restraints of the moral law, and of its entire faith. Many other causes were in operation, of much too complex a nature to be analysed here; but the combination of these two elements clearly indicates that the lack *was not* in the deficiency of intellectual energy, and *might be* in the moral want. And with the thunder of the French Revolution still sounding in our ears, Mr. Buckle would assert that the literature of the Encyclopædia was "merely the record of the knowledge" of the people.

But we will cease our refutation of these flimsy paradoxes, and take our leave of Mr. Buckle with a cordial expression of our admiration for his vast learning and ability, and an undoubting trust that of the many who will be charmed with the literary merits of his work, but few will adopt his philosophy; but, above all, with an earnest hope that in later years he himself may be able to borrow a *mot* from a notorious character of our country, and, for the sake of truth and religion, be able to confess that he is not a Buckle-ite.*

The benefits of civilization are found in the improved moral, physical, and intellectual condition of a people, and the mode in which these improvements manifest themselves are patent to all; we need not weary onr readers by recapitulating them. But these benefits are not without alloy. It seems to be a law of society that the happiness of the many shall involve the wretchedness of a few. Whilst comfort and riches are diffused on a more graduated plan, the more highly civilized is the community; yet it seems to admit, exceptionally, of greater misery amongst certain classes than even some forms of savage life. And this must continue to be the case so long as civilization is a state of *becoming*, and not *being*.

Civilization also introduces into society certain sources of sudden alternations of prosperity and adversity, which are unknown in savage life. An illustration of this is found in the tendency to speculation, of which such frightful examples have been before us of late years. On these and analogous points we need not dwell.

* John Wilkes, in his later years, being asked by the King after one of his former friends, replied, "Oh, please your Majesty, don't call him a friend of mine; he was a Wilke-ite—*I never was.*"

M. Guizot, in his eloquent introductory lecture on the "History of Civilization in Europe," acknowledges that one of the questions which may be asked about civilization is, whether it is a good or an evil—"for some bitterly deplore it, some rejoice at it." Although we do not hesitate in any degree to agree with him as to the magnitude of the blessings of civilization to the world, yet we see that in the transition periods evils of gigantic character do occur. Our limits compel us to confine ourselves to a brief notice of only two of these. The first is the effect of the contact of civilized with savage life; the second is concerning the great increase of mental maladies proportionate to the advance of civilization.

The disappearance of entire races of men before the civilizing influences of the white man, is an established fact. It has been observed even in Europe, it is notorious in America, and also in Australia to a smaller extent. This is due to many and complicated causes. We introduce diseases amongst them to which they were strangers before, such as the small-pox, which has swept away whole tribes of the North American Indians. We introduce amongst them gunpowder and alcohol, in their effects as deadly as the small-pox. We cultivate their land; and this process once begun has no limits to the Teutonic race, save the nature of the soil and the climate. The native is driven before us, until one or other of these influences stops our progress. In America these results are very rapid; from the Atlantic to the Appalachian system, scarcely any vestige of the red man is to be found; from the latter to the Mississippi the same results are rapidly approaching. The red man is fast disappearing before the aggressions of his white brother; and in other parts of the world a similar process is taking place.

Yet, though these results emanate from civilized lands, they cannot be fairly considered the products of civilization, but rather of the *want* of that *true civilization* which, as we have been endeavouring to show, depends upon a due recognition of the moral law, keeping strict pace with the intellectual development. It is because there is not true love and charity and brotherhood between man and man, that these inhuman encroachments upon native privileges occur. And of these we shall see no more, should the time ever happily arrive when civilization is firmly established upon the foundation of the moral law, which for one of its great roots has the command to "love our neighbour as ourselves." Yet, before that time arrives, it is to be feared that an interesting race of people will have vanished from the face of the earth, whom we may mourn, but cannot restore.

The second and last point connected with the evils attendant upon civilization which we propose to notice is, the very great increase of insanity as civilization progresses. The following Table shows how great is the proportion of insane persons to the whole population in the most civilized cities, compared with that in those less so. The numbers refer to the population of some years back:—

| | Population. | No. of Insane. | Proportion. |
|---|---|---|---|
| London | ...1,400,000 | ... 7000 | ... 1 in 200 |
| Paris ... | ... 890,000 | ... 4000 | ... 1 „ 222 |
| Milan ... | ... 150,000 | ... 618 | ... 1 „ 242 |
| Florence | ... 80,000 | ... 236 | ... 1 „ 338 |
| Turin ... | ... 114,000 | ... 331 | ... 1 „ 344 |
| Dresden | ... 70,000 | ... 150 | ... 1 „ 466 |
| Rome ... | ... 154,000 | ... 320 | ... 1 „ 481 |
| Naples... | ... 364,000 | ... 479 | ... 1 „ 759 |
| Petersburgh.. | 377,046 | ... 120 | ... 1 „ 3142 |
| Madrid | ... 201,000 | ... 60 | ... 1 „ 3350 |
| Cairo ... | ... 330,000 | ... 14 | ... 1 „ 23,571 |

It is not unlikely that there may be some error in the formation of some of these statistics; but with all allowance made for error, it is clear that the principal seats of civilization are those where insanity is most rife.

M. Brierre de Boismont, in a memoir upon "The Influence of Civilization on the Development of Insanity," comes to the following conclusions:—

1. Insanity is more frequent in proportion as civilization is more developed, and is more rare where the people is less enlightened.

2. Amongst the former, insanity is due to moral causes; amongst the latter, almost exclusively to physical causes.

3. A like distinction is observed in the civilized and uncivilized classes in the same community. Amongst the former, insanity is chiefly due to moral, in the latter to physical causes.

4. Every age and every country witnesses the origin of forms of insanity bearing the impress of the dominant ideas of that age or country, and having the seal of the epoch.

5. Every remarkable event, every grave public calamity, augments the number of the insane.

6. The increase of insanity is in relation to the development of the intellectual faculties, of the passions, of industry, of riches, and of misery.

7. As the amount of insanity is strictly in proportion to the amount of civilization, and is determined in great part by moral causes; moral means—especially those which will exercise a mild regulating influence over the passions—will form the principal basis of cure, especially in convalescence. And the chances of restoration will be the greater in proportion as the patients are better instructed, and the social classes more enlightened. But as for this the strictest *surveillance* is requisite, the best results are to be expected from well-regulated and numerous establishments, founded and carried on upon these principles.

We will conclude this subject by some observations of Dr. Feuchtersleben, which fully express our sentiments on the cause of this effect of civilization on insanity:—

"A practical proof of the morbific power of the emotions and passions is found in the frequent occurrence of psychopathies in times when all the elements of social life are in a state of fermentation; in and after revolutions, when sudden changes of fortune, loss of property, worldly elevation and depression, fill the lunatic asylums, and (if Pariset be right) produce a thousand cases of mental disorder which, in the general turmoil, remain unknown and unmentioned. And herein lies the answer to the question, why the number of mental diseases has increased with civilization?—a question which has certainly proved to be a fact. It is not civilization, but the increasing want which it brings in its train,—partial education, passions, emotions, &c., all which set the mind in passive motion; the forced culture to which they lead; the over-indulgence,—these contain the reasons of the fact. Civilization, as external education, is but a transition to culture as internal education; and in this first stage it produces evils for which it furnishes the remedy in the higher stages. It carries the poison and the antidote in the same hand."

Thus have we attempted to trace the nature, progress, results, advantages, and evils of civilization. At some future time we propose to indicate the application of some of these principles to the regeneration of the fallen and dangerous classes of civilized societies.

---

